# Comparative Effects of Blood Flow Restriction and Traditional Strength Training on Proximal Shoulder Musculature: A Randomized Clinical Trial

**DOI:** 10.3390/muscles4030034

**Published:** 2025-08-18

**Authors:** Lucas Ghionna, Léa Ruppel, Nuno Nogueira, Gabriela Brochado, Alice Carvalhais

**Affiliations:** 1 Departamento de Tecnologias de Diagnóstico e Terapêutica, Escola Superior de Tecnologias da Saúde do Tâmega e Sousa, Instituto Politécnico de Saúde do Norte (IPSN), Cooperativa de Ensino Superior Politécnico e Universitário, 4585-116 Gandra, Portugal; lucas.ghionna@gmail.com (L.G.); learuppel57@gmail.com (L.R.); nuno.nogueira@ipsn.cespu.pt (N.N.); gabriela.brochado@ipsn.cespu.pt (G.B.); 2 Center for Rehabilitation Research—Center of Human Studies and Human Activity, 4200-072 Porto, Portugal; 3 Health and Human Movement Unit, Polytechnic University of Health, Cooperativa de Ensino Superior Politécnico e Universitário, 4760-409 Vila Nova de Famalicão, Portugal; 4 Laboratório Associado em Energia, Transportes e Aeroespacial, Instituto de Ciência e Inovação em Engenharia Mecânica e Engenharia Industrial, Faculty of Engineering, University of Porto, 4200-465 Porto, Portugal

**Keywords:** endurance, female, hypertrophy, KAATSU, male, power, shoulder, strength

## Abstract

Background: Blood Flow Restriction (BFR) training may be an alternative when traditional heavy-load training is unsuitable. This study compared BFR with light loads to traditional strength training for shoulder muscle development proximal to the occlusion site; Methods: A total of 22 healthy adults were randomized into Group A: BFR training (30% 1RM; *n* = 12) and Group B: Traditional strength training (70% 1RM; *n* = 10). Four-week protocol (2 sessions/week) included shoulder abduction and lateral rotation, and dumbbell overhead press. Arm circumference, Single Arm Seated Shot-Put Test (SAASPT), vertical lift strength (VLS) and Shoulder Endurance Test were assessed at baseline and at the end of the protocol. Cohen’s d effect size was calculated for significant outcomes; Results: Significant gains occurred in both groups across most parameters. The magnitude of effects was, in Group A, large on Arm circumference and SASSPT (Cohen’s d = 0.870 and 1.158, respectively) and very large in VLS and SET (Cohen’s d = 1.284 and 1.301, respectively). In Group B, the magnitude of effects was large in SASSPT and VLS (Cohen’s d = 0.962 and 0.922, respectively) and very large in SET (Cohen’s d = 1.238); Conclusion: BFR training with light loads effectively improved musculature proximal to the occlusion site, demonstrating comparable strength gains to heavy-load training in healthy individuals.

## 1. Introduction

Therapeutic exercise overload is widely recognized as the most effective and prevalent approach in the rehabilitation of the musculoskeletal system across various regions of the human body. Evidence-based guidelines for muscle strengthening protocols, such as those proposed by the American College of Sports Medicine (2009) [1], emphasize the principle of physiological adaptation to mechanical stress. These protocols typically prescribe varying load intensities, with hypertrophy-oriented regimens recommending loads ranging from 70% to 80% of an individual’s one-repetition maximum (1RM) [1]. However, such protocols are often impractical in clinical contexts where patients present with limitations such as pain, structural deficits, or post-surgical recovery. This situation poses significant challenges for physiotherapists, particularly in managing shoulder pathologies, which rank among the most prevalent musculoskeletal disorders of the upper limb [2]. These conditions affect a wide range of individuals, including both athletes [3] and non-athletes [2], thereby requiring the development of adaptable and effective rehabilitation strategies. In many clinical scenarios, reaching the high load thresholds needed to stimulate muscle hypertrophy or maintain strength is often unfeasible, particularly in postoperative settings or when pain, muscle atrophy, or structural damage is present. Over the past decade, Blood Flow Restriction (BFR) training has garnered increasing attention for its proven efficacy in enhancing muscle strength and hypertrophy, even under low-load conditions [4]. Initially developed by Yoshiaki Sato in 1966 in Japan [5], BFR training involves performing low-load exercises (30% of 1RM) while restricting blood flow through a compressive cuff applied at the proximal limb [4]. Although the precise mechanisms remain incompletely understood, studies suggest that BFR induces metabolic stress through venous occlusion and partial arterial flow restriction [6]. These conditions activate various cellular pathways, promoting enhanced muscle strength and hypertrophy. Specifically, hypoxic and acidic intramuscular environments stimulate cellular growth, proliferation, and protein synthesis [7]. Furthermore, BFR facilitates angiogenesis as a compensatory response to hypoxia [8]. The metabolic stress induced by BFR training preferentially recruits type II muscle fibers [9].

To date, BFR training has been investigated more extensively in the lower limb than in the upper limb. Moreover, most research in this field has primarily focused on the distal effects relative to the occlusion site. However, recent evidence suggests that muscles proximal to the occlusion, such as the shoulder muscles, also derive significant benefits in terms of strength and hypertrophy [10]. Several mechanisms have been proposed to explain these proximal adaptations, including remote ischemic preconditioning, a backflow phenomenon where vascular pressure builds proximal to the cuff [6], and increased EMG activity of proximal musculature [11].

Despite these promising findings, significant knowledge gaps remain regarding the efficacy of upper limb BFR training for shoulder muscle development. Specifically, this study compared the effects of BFR training versus traditional high-load training on comprehensive shoulder muscle function, including strength, power, endurance, and morphological adaptations. Furthermore, the optimal BFR protocols for shoulder muscle training remain undefined, limiting clinical translation and evidence-based implementation.

Thus, we hypothesize that vascular restriction applied to the upper limb, in combination with low-load exercises, can effectively enhance shoulder muscle hypertrophy, power, endurance, and strength. This study aimed to compare the effects of an 8-week BFR training program using 30% of one-repetition maximum (1RM) versus traditional strength training using 70% of 1RM on shoulder muscle strength, power, endurance and hypertrophy in healthy adults. Demonstrating these effects in a healthy population could provide critical insights into advancing clinical approaches to shoulder rehabilitation, particularly in scenarios where the use of high loads is contraindicated or impractical.

## 2. Results

**Figure 1:**
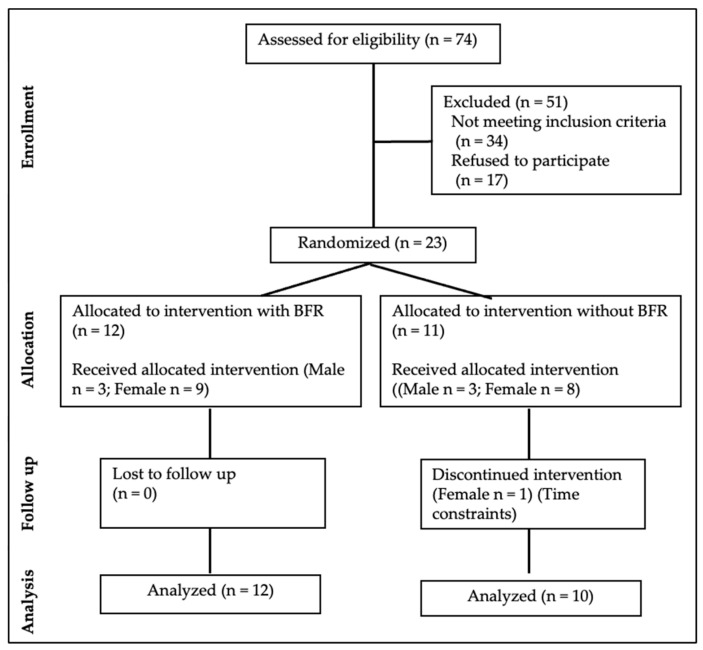
Flowchart of participant enrolment, allocation, follow-up and analysis.

Seventy-four individuals responded to the questionnaire and forty were eligible to participate in the study. Twenty-three accepted to participate in the study, twelve in the BFR group and eleven in the High-load group (without BFR). One participant in the High-load group dropped out after two sessions (Figure 1).

Participants’ characteristics by group are presented in Table 1.

No differences in age, BMI or sex were found between groups (Table 1).

No differences in arm circumference (ArmC), SASSPT (Single Arm Seated Shot-Put Test), vertical lift strength (VLS), or Shoulder Endurance Test (SET) were found between groups, either at baseline or at the end of the protocol (Table 2).

In ArmC, a significant increase was observed only in BFR group. Regarding the remaining tests, the increases were significant in both groups. The magnitude of the phenomena was higher in all outcome measures in BFR group. Thus, in BFR group, the effect size was large in ArmC and SASSPT, and very large in VLS and SET. As for High-load group, the effect size was large in SASSPT and VLS, and very large in SET (Table 3).

Post-session muscle soreness (Delayed Onset Muscle Soreness) and training discomfort were the most frequently reported symptoms in the BFR group. Discomfort was typically experienced during the final repetitions of each set (30/15/15/15), especially during initial sessions, but consistently scored below 7/10 on a numeric scale as recommended [12,13]. These symptoms diminished over subsequent sessions, with reports of muscle soreness ceasing after the second week. No adverse events or complications were recorded throughout the study period.

## 3. Discussion

The aim of this study was to compare the efficacy of a training protocol through the application of BFR on the upper limb to traditional strength training, on the proximal muscles of the shoulder proximal to the occlusion site, in healthy adults. Both training types improved muscle strength, power and endurance, but the magnitude of the effect was slightly higher on the BFR training group.

The findings of the present study align with emerging evidence suggesting that proximal musculature can benefit from the metabolic effects induced by this technique [11,14].

Most studies on BFR application in the upper limb focused on its distal effects to the occlusion region [15,16]. However, our results support the growing body of evidence demonstrating proximal benefits. A recent systematic review and meta-analysis reported that low-load BFR training seems to be as effective as high-load resistance training in improving strength in upper body muscles proximal to the occlusion site in healthy adults [10]. This finding is further supported by a recent study, which likewise demonstrated comparable strength gains under similar conditions [17]. Supporting evidence for this phenomenon includes a study demonstrating significant hypertrophy of the pectoralis major following low-intensity bench press training with BFR [18].

The superior results observed in the BFR group can be attributed to several interconnected mechanisms. BFR training has been shown to enhance motor unit recruitment, thereby promoting neuromuscular adaptations in proximal musculature. For example, Fatela et al. [19] reported increased activation of motor units with higher action potential amplitudes during low-intensity resistance exercises with BFR. This effect was corroborated in a recent study by Liu et al. [20], highlighting the ability of BFR to activate motor units typically engaged during high-intensity exercises, even at lower loads. These neurological adaptations may explain the strength improvements obtained in the present study, particularly given the relatively short intervention duration.

From a metabolic perspective, the enhanced efficacy of BFR can be attributed to the creation of a localized hypoxic environment, which stimulates the upregulation of cellular signaling pathways [6]. Additionally, BFR is known to preferentially recruit type II muscle fibers through metabolic stress induced by occlusion [9]. Interestingly, a recent systematic review revealed equivalent hypertrophy in both type I and type II fibers with BFR training, suggesting that fiber activation patterns may be broader than previously thought. These findings underscore the need for further research to better understand the mechanisms underlying endurance improvements with BFR strength training [21].

Both groups exhibited significant strength gains in the shoulder muscles proximal to the occlusion site. While favorable outcomes were anticipated for the traditional High-load training group, known to promote muscle hypertrophy via mechanical stress and subsequent strength adaptations [1], the BFR group achieved superior results in terms of effect size. These findings reinforce the potential of BFR training to elicit neuromuscular improvements across both proximal and distal muscle groups through the mechanisms described above.

Both groups showed significant improvements in power test performance, with similar effect sizes between groups, though slightly higher in the BFR group. As power is a function of strength and speed, this improvement may reflect the observed strength gains. Since both strength and power showed similar patterns of improvement between groups, with slightly greater effect sizes in the BFR group, this suggests a consistent adaptation pattern. However, as speed was not independently assessed, further studies are needed to elucidate the specific contributions of strength and speed to power enhancements.

Although the exercises implemented were not designed to develop muscular endurance, significant improvements in this parameter were observed in both groups. Despite traditional high-load hypertrophy training and BFR with low-load training being associated with muscle mass increases [1,14], ArmC increased significantly only in the BFR group. This improvement is consistent with the greater magnitude of effect on maximal strength observed in this group. ArmC is a non-invasive, widely used indirect measure of muscle mass [22], though it encompasses other tissue components, such as fat and fluid, which could bias our results. To minimize measurement error, consistent methodologies and evaluator training were employed. These changes could reflect transient edema and fluid shifts rather than true hypertrophy, given that the protocol lasted only 4 weeks.

The findings of the present study suggest that both high-load resistance training and BFR low-load resistance training elicit measurable improvements in muscular strength, power, and endurance, particularly in the trained musculature proximal to the pressure site. Given the relatively short duration of the intervention (4 weeks), it is reasonable to infer that the performance gains observed are primarily mediated by neural adaptations, rather than structural changes, such as muscle hypertrophy or fiber-type transitions, which typically require longer periods of training to manifest [23].

Current evidence regarding long-term differences in muscle excitation between high-load and low-load BFR training remains inconclusive. However, central activation, assessed through electrically evoked superimposed twitches, has been shown to increase significantly following high-load training, whereas low-load BFR does not consistently elicit the same response. EMG data from prior studies further support the notion that low-load BFR may induce neural adaptations comparable to those elicited by high-load training [24]. However, these findings are limited to distal musculature, that is, the muscle groups directly involved in the exercise and located downstream from the site of vascular restriction. It remains unclear whether similar neural adaptations occur in proximal muscle groups, where the hemodynamic and neuromuscular responses to BFR may differ. Moreover, it remains unclear whether the neural adaptations reported in longer term interventions also occur following shorter training duration, such as the 4-week period implemented in the present study. Future studies incorporating both distal and proximal measurements, alongside advanced neurophysiological assessments, are warranted to address this gap.

Participants in this study did not engage in sports involving intensive use of the dominant upper limb, such as tennis, volleyball, or handball. Those who reported regular or irregular physical activity (e.g., running, swimming, or fitness) limited participation to less than 3 h per week. The potential for improvement is generally greater in untrained individuals compared to those already trained, which may partially explain the observed increases over the 4-week protocol [1]. The previous literature indicates that the initial benefits of BFR training become evident after 4 weeks of twice-weekly sessions [25], with more pronounced effects emerging with longer training durations. Extending the protocol to 6–8 weeks or more could enable load progression based on individual performance, potentially yielding even greater improvements. A longer study duration would also allow for intermediate evaluations, including recalibration of 1RM.

From a clinical perspective, the findings of this study indicate that low-load resistance training combined with BFR is sufficient to elicit significant improvements in strength, power, and endurance in the shoulder complex. This technique emerges as a promising and adaptable tool within rehabilitation settings, offering multiple advantages, particularly in cases where high-load resistance training exercises are contraindicated or when optimizing functional performance is prioritized. Therefore, BFR training with low loads can be considered a viable and effective alternative to traditional high-load training for shoulder muscle strengthening in healthy individuals.

The limitations of this study should be acknowledged. The absence of blinding among participants and researchers may have introduced bias, as superior outcomes in the BFR group could be expected. Lifestyle factors such as diet, sleep quality, and stress levels were not controlled, which may have influenced outcomes. Adherence to the recommendation to maintain consistent physical activity levels throughout the study could not be independently verified, although participants were instructed to do so. Despite the acknowledged limitations, the implementation of an automated, validated BFR system for occlusion pressure determination represents a significant advantage. Unlike studies relying on manual pressure estimation or fixed percentage protocols, our automated approach eliminated potential bias in pressure determination and ensured consistent, reproducible occlusion pressures across all assessment timepoints and training sessions. This standardized methodology mitigates concerns regarding measurement variability and enhances the reliability of between-group comparisons.

Although the findings of the present study suggest that low-load BFR training produces effects equivalent to those of traditional high-load resistance training on shoulder muscle strength, power, and endurance, it remains unclear whether these outcomes are attributable to any single proposed mechanism, such as localized hypoxia-induced metabolic stress, hormonal responses, potential hemodynamic changes proximal to the occlusion site and increased neuromuscular activation, or rather to a synergistic interplay of multiple factors [6]. Further studies employing more targeted physiological assessments are needed to elucidate the specific pathways responsible for the adaptations observed with BFR training.

This study opens opportunities for future research. While cross-transfer effects to the contralateral limb were not assessed, previous studies demonstrated such effects in non-BFR-trained limbs [4,26], warranting investigation of homolateral upper limb adaptations. Future studies should strive for balanced gender representation, as limited research has addressed potential differences in muscle strength, hypertrophy, endurance, and power between males and females [27]. Mechanistic studies could elucidate gender-specific responses to BFR training. Additionally, research should explore BFR training at similar intensities (30% 1RM) with varied training contexts, such as increased session frequency or reduced recovery periods, to optimize metabolic stress. Finally, investigating BFR utility in specific populations, including those with pathologies or different age groups, remains important for future work.

## 4. Materials and Methods

### 4.1. Study Design

This investigation was designed as an experimental randomized clinical trial (RCT) and adhered to the guidelines outlined in the Consolidated Standards of Reporting Trials (CONSORT) statement [28] (Figure 1). All the subjects provided written informed consent in accordance with the Declaration of Helsinki. This study was approved by the ethics committee of Instituto Politécnico de Saúde do Norte (31/CE-IPSN/2024).

### 4.2. Sample

Healthy individuals aged 18 to 40 years were recruited for the study through social media, discussion groups, and physical postings at CESPU facilities. Participants of both sexes were included, as previous studies have demonstrated comparable metabolic responses to BFR across genders [29]. A body mass index (BMI) between 18.5 and 30 was required, as individuals with obesity are frequently excluded from such studies due to an increased risk of cardiovascular, respiratory, and rheumatologic complications. Exclusion criteria included participation in sports that heavily involve the dominant arm (e.g., tennis, volleyball, or handball) and do not engage in strength training exercises on the upper limb, a history of trauma or surgery affecting the dominant upper limb, acute or chronic shoulder pain, radiating pain, cervical disc herniation, previous neck surgeries, or upper limb edema. Additional exclusion factors were a history of deep vein thrombosis, oncologic or metabolic conditions, such as diabetes, and pregnancy. Participants were also excluded if they experienced persistent symptoms such as numbness, tingling, or pain exceeding 7/10 on a numeric scale during the training (applicable to BFR group) or if they missed more than one session during the study.

Participants were randomly assigned using a simple randomization tool provided by the Sealed Envelope™ (https://www.sealedenvelope.com, accessed on 1 July 2025) website: BFR group, which followed a protocol with BFR and low load (30% 1RM), and High-load group, which adhered to a traditional high-load protocol (70% 1RM).

### 4.3. Procedures

Participants’ eligibility was assessed by an online questionnaire comprising personal data, sports activity, and medical history questions. Eligible individuals were contacted via email to schedule the first evaluation session. Before starting the study, a familiarization period was conducted to train researchers on using equipment, administering tests, and applying training protocols. Clear instructions and safety procedures were emphasized, ensuring consistency in application and measurement accuracy. Each researcher was assigned specific tasks, and adjustments were made as necessary.

For all participants, measurements were conducted after a warm-up following the same order: ArmC, SASSPT, VLS and SET. Measurements were performed at baseline (M0) and at least 24 h after the last training session (M1). Participants were instructed to avoid any intensive upper limb physical effort in the 24 h before testing.

#### 4.3.1. Training Protocol

The study protocol lasted four weeks, with two sessions per week. Each session began with a warm-up, including mobility and stretching exercises for the shoulders and upper limbs. At the first session 1RM was determined for each participant and exercise, using a failure-to-repetition method with applied coefficients [12].

**Figure 2:**
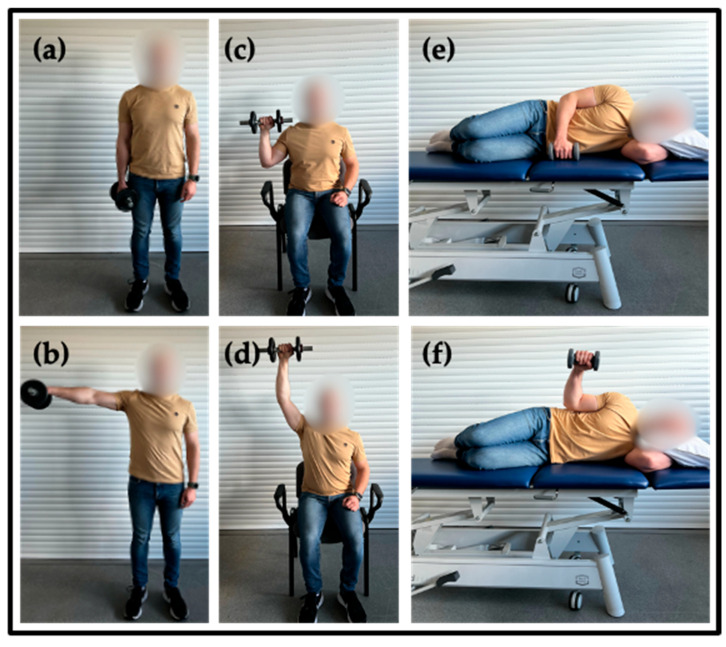
Protocol exercises (**a**) Shoulder Abduction initial position; (**b**) Shoulder Abduction final position; (**c**) Dumbbell Overhead Press initial position; (**d**) Dumbbell Overhead Press final position; (**e**) Shoulder Lateral Rotation initial position; (**f**) Shoulder Lateral Rotation final position.

Three exercises which targeted the shoulder musculature were included:

(1) Performed in a standing position, raising the upper limb to 90° of abduction and then returning to the starting position. (Figure 2a,b).
(2) Dumbbell Overhead Press: Executed seated with back support. Weight was elevated vertically from shoulder level to above the head and returning (Figure 2c,d).
(3) Shoulder Lateral Rotation: Performed lying on a bench. Participant was seated with the arm resting against the torso and the elbow flexed at 90°. The movement involved performing shoulder external rotation (Figure 2e,f).

High-load group: Training followed ACSM guidelines for hypertrophy, at 70% of 1RM [12]. Four sets of 8 to 10 repetitions were completed for each exercise, with 2-min rest between sets and exercises. Movement speed was moderate (1-s concentric, 2-s eccentric).

BFR group: Exercises were performed, with 30% 1RM, in a specific sequence, totaling 75 repetitions (4 sets—30/15/15/15, with 30-s rest intervals between sets [4,12,25]). Movements were performed at a 4-s tempo (2 s concentric, 2 s eccentric).

The cuff pressure was maintained throughout the exercise, released for 60 s between exercises, and reapplied for the next exercise. Participants rated exercise difficulty, including pain, tension, and numbness, using a numeric scale from 0 (no symptoms) to 10 (unbearable). Symptoms exceeding 7/10 on a numeric scale prompted adjustments in protocol or session cancellation.

Each training session lasted approximately 45 min for both groups. Participants were advised to maintain the same physical activity levels during the study.

##### Determination of Blood Flow Restriction Pressure (BFR)

The Smartcuffs^®^ 3.0 PRO standard set by Smart Tools Plus was used for BFR application. The device automatically determined arterial occlusion pressure (AOP) values at the beginning of each session, applying a limb occlusion pressure (LOP) of 50% [30]. Participants were seated, and the cuff (7 cm width) was placed directly on the skin or over a thin T-shirt at the uppermost part of the dominant arm (proximal to the deltoid insertion) [12].

#### 4.3.2. Data Collection

##### Arm Circumference

The ArmC at the armpit level of the dominant limb was considered representative of the deltoid muscle mass. The measurement was performed at rest using a standard measuring tape. The average of three measurements was used for analysis.

##### Muscle Power

**Figure 3:**
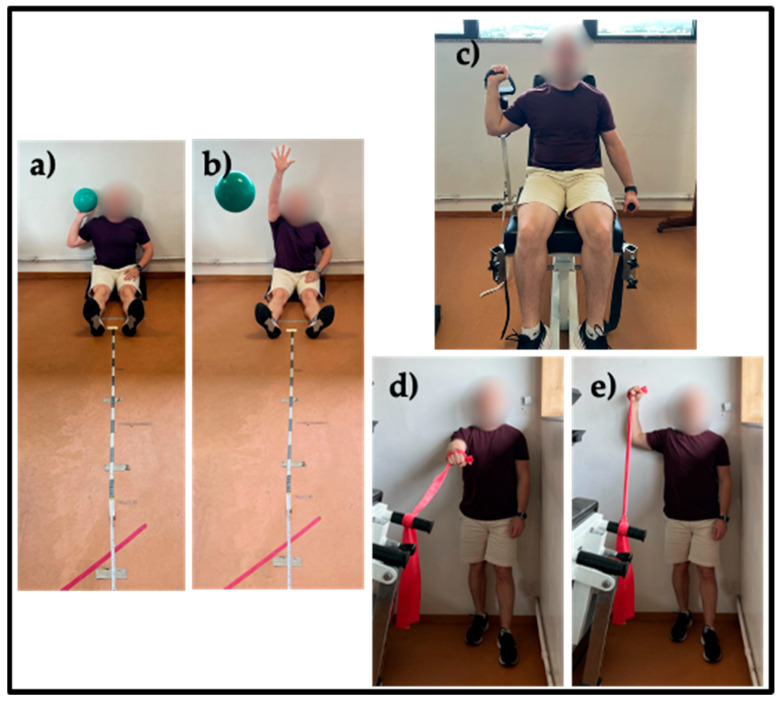
Outcomes measurements (**a**) Single Arm Seated Shot-Put Test initial position; (**b**) Single Arm Seated Shot-Put Test final position; (**c**) vertical lift strength test position; (**d**) Shoulder Endurance Test initial position; (**e**) Shoulder Endurance Test final position.

The Single Arm Seated Shot-Put Test (SASSPT) was chosen to assess shoulder muscle power and upper limb functionality. Participants sat with their trunk and shoulders against a wall, lower limbs extended, and the non-tested arm placed on the ipsilateral leg to avoid compensation [31]. Participants threw a 4 kg medicine ball as far as possible. Distances were recorded using floor-placed tape measures, with video recording to control ball contact points. Three measurements were taken, with the average used for analysis (Figure 3a,b).

##### Vertical Lift Strength

The Smart Groin Trainer (NeuroExcellence) (Braga, Portugal) was used to measure isometric muscle strength, focusing specifically on a vertical lift task. Concurrent validity and reliability have been demonstrated with r > 0.89 and ICC coefficients between 0.94 and 0.99, respectively [32]. Isometric muscle strength was evaluated with participants seated, the shoulder positioned at 60° of abduction, the elbow in flexion, and force applied in the direction of vertical arm elevation. Participant performed three attempts, and the best result was considered for analysis [11] (Figure 3c).

##### Shoulder Endurance Test

The Shoulder Endurance Test (SET) was chosen to evaluate shoulder muscle endurance due to its controlled pace. Participant stood with his back against a wall, the contralateral heel touching the wall, and the ipsilateral leg positioned forward. Movement involved abducting the shoulder to 90° and externally rotating to 90° while touching the wall. Resistance was applied using a red elastic band. The distance between the hand and elastic anchor was fixed at 1 m. A metronome was used to guide movement cadence from 60 bpm, increasing by 30 bpm every 20 s, until 150 bpm. The test ended when participants failed to maintain pace or movement quality [33]. Test duration was recorded in seconds (Figure 3d,e).

#### 4.3.3. Data Analysis

Statistical analysis was conducted using SPSS (version 29.0, SPSS Inc., Chicago, IL, USA). Quantitative variables were described as means and standard deviations or median and interquartile range as appropriate. Group comparisons employed Fisher’s exact test, Mann–Whitney U-test, or independent sample *t*-tests. Paired sample *t*-tests compared pre- and post-intervention results. Cohen’s d effect size was calculated for significant outcomes and classified as Very small: 0.01 < d ≤ 0.20, Small: 0.20 < d ≤ 0.50, Moderate: 0.50 < d ≤ 0.80, Large: 0.80 < d ≤ 1.20, Very large: 1.20 < d ≤ 2.00 and, Huge: d > 2.00. A significance level was set at *p* < 0.05.

## 5. Conclusions

Blood Flow Restriction (BFR) training with light loads could be considered an equivalent method to heavy-load training for shoulder strengthening in healthy individuals. The findings from this study confirm the efficacy of the applied protocol using low load to effectively strengthen the shoulder complex. This technique holds promise as a versatile rehabilitation tool, offering multiple benefits and is particularly well-suited to scenarios where high-load exercises are not feasible or when performance optimization is the goal.

## Figures and Tables

**Table 1:** Participants’ characteristics.

		BFR Group (*n* = 12)	High-Load Group (*n* = 10)	*p*
		N (%)	N (%)	
Sex	Female	9 (75)	7 (70)	0.583 ^a^
	Male	3 (25)	3 (30)	
		x̅ ± sd (min–max)	x̅ ± sd (min–max)	
Age (years)		22.3 ± 1.6 (19–25)	22.6 ± 2.1 (20–26)	0.659 ^b^
BMI * (kg/m^2^)		23.5 (1.9)	22.4 (4.2)	0.598 ^c^

BFR, Blood Flow Restriction; BMI, Body mass index; ^a^ Fisher exact test; ^b^ *t*-test independent samples; ^c^ Mann–Whitney test; * Mean and interquartile range.

**Table 2:** Comparison of arm circumference, power, strength, and endurance between groups at baseline and final assessments.

	M0			M1		
	BFR Group	High-Load Group		BFR Group	High-Load Group	
	x̅ ± sd		*p*	x̅ ± sd		*p*
ArmC (cm)	31.97 ± 2.02	31.92 ± 4.05	0.974 ^a^	32.91 ± 2.41	32.49 ± 3.23	0.270 ^a^
SASSPT (cm) *	250.00 (83)	247.50 (99)	0.273 ^b^	280.00 (93)	269.50 (110)	0.531 ^b^
VLS (kg)	18.95 ± 7.45	19.22 ± 9.74	0.942 ^a^	24.47 ± 9.73	24.10 ± 12.71	0.406 ^a^
SET (s)	62.08 ± 12.73	59 ± 13.47	0.588 ^a^	95.67 ± 31.33	78.50 ± 22.25	0.303 ^a^

ArmC, Arm circumference; BFR, Blood Flow Restriction; M0, baseline assessment; M1, final assessment; SASSPT, Single Arm Seated Shot-Put Test; SET, Shoulder Endurance Test; VLS, vertical lift strength; ^a^ *t*-test independent samples; ^b^ Mann–Whitney test; * Median and interquartile range.

**Table 3:** Comparison of mean differences in arm circumference, power, strength, and endurance between baseline and final assessments, along with corresponding effect sizes, for both groups.

	BFR Group (*n* = 12)			High-Load Group (*n* = 10)		
	M1−M0 x̅ ± sd	*p* ^a^	Cohen’s d	M1−M0 x̅ ± sd	*p* ^a^	Cohen’s d
ArmC (cm)	0.94 ± 1.08	0.012	0.870	0.57 ± 1.25	0.182	0.457
SASSPT (cm)	19.67 ± 16.98	0.002	1.158	22.00 ± 22.88	0.014	0.962
VLS (kg)	5.52 ± 4.30	<0.001	1.284	4.88 ± 5.30	0.017	0.922
SET (s)	33.58 ± 25.82	<0.001	1.301	19.50 ± 15.76	0.004	1.238

ArmC, Arm circumference; M0, baseline valuation; M1, final evaluation; SASSPT, Single Arm Seated Shot-Put Test; SET, Shoulder Endurance Test; VLS, vertical lift strength; ^a^ *t*-test paired samples.

## Data Availability

Data will be available upon reasonable request from the corresponding author.

## Abbreviations

The following abbreviations are used in this manuscript:

BFR	Blood Flow Restriction
BMI	Body Mass Index
SASPT	Single Arm Seated Shot-Put Test
SET	Shoulder Endurance Test
VLS	Vertical Lift Strength
